# Combined e-pharmacophore based screening and docking of PI3 kinase with potential inhibitors from a database of natural compounds

**DOI:** 10.6026/97320630015709

**Published:** 2019-10-20

**Authors:** Sasidhar Reddy Eda, Rajeswari Jinka

**Affiliations:** 1 Department of Biochemistry, Acharya Nagarjuna University, Guntur, Andhra Pradesh, India

**Keywords:** PI3-kinase, screening, docking studies, MM-GBSA

## Abstract

Phospho inositide 3-kinase (PI3 K) is a promising target for the design of anticancer drugs and is of significant concern in developing selective isoforms as inhibitors for cancer treatments. The results obtained from the computational analysis were selected based on Glide score and drug binding interaction features. Molecular docking studies and prime MM-GBSA energy calculations showed STOCK1N-77648 with optimal binding features for further consideration. The hydrogen bonding patterns between the top three molecules STOCK1N-91335, STOCK1N-70036 and STOCK1N-77648 and the target protein based on G-scores is reported. The STOCK1N-77648 ligand molecule has protein residue interactions similar to that of interactions with the known inhibitor copanlisib. These data illustrates selectivity of the small molecular PI3 K inhibitors through screening and molecular docking for further in vitro and in vivo consideration.

## Background

Tumor development and invasion are not only the consequence of malignant transformation; they also depend on surrounding stroma environmental influences, local growth factors, and systemic hormones. It is believed that the composition of the extra cellular matrix (ECM) affects malignant behaviour that may depend on the differentiation status of tumor cells [1]. Recent studies emphasize the importance of extra cellular matrix in cell life and death decisions. The local status of the ECM may be profoundly disturbed by inflammation and tumorigenesis. Tumors can secrete in abundance various ECM components and metallo proteases, thereby altering the immediate tissue environment [2].

Integrins are αβ-transmembrane protein hetero dimers whose non-covalent association define the specificity of adhesion to specific ECM elements or alternative proteins that sense the cell micro-environment and modulate numerous signalling pathways. Changes in integrin expression between normal and tumor cells promote tumor development and aggressiveness involvement of specific integrins. α5β1 integrin merits interest in colon, breast, ovarian, lung and brain tumors where their over expression is related with a poor prognosis for patients. Few studies highlight a crucial function of β1-integrin in the metastatic dissemination of tumor cells and in cellular senescence [3,4]. Higher expression of α3, α5, α6, αv, β1, β4, α6β4, α9β1, αvβ5 and αvβ3 integrins are directly associated with the disease progression. Several epithelial tumors showed altered integrin expression of α6β4, α6β1, αvβ5, α2β1 and α3β1. Tetra spanins have been shown to regulate integrin recruitment into membrane micro domains and crucially regulate integrin characteristic in tumor cells. Recent studies have shown that cell signalling in transformed cells generated with the aid of growth factors and oncogenes requires collaboration with specific integrins, particularly during initiation of the tumour. In tumor cells, multiple survival signals are upregulated on integrin ligation, which includes increased expression of BCl-2 or FlIP (also known as CflAR), activation of the PI3K-AKT pathway or nuclear factor-µB (nF-µB) signalling, and/or p53 inactivation [5].

Integrin engagement with the ECM molecule was shown to be directly mediated by AKT activation via PI3K signalling through the direct recruitment of PI3 K into the subunit [6]. Phosphatidyl inositol 3-kinase (PI 3-kinase) enzyme catalyzes the phosphorylation of inositol lipids at the D-3 position of the inositol ring, ensuing in the formation of the 3-phosphorylated phosphoinositides (3-PPIs): phosphatidyl inositol 3-phosphate [PI(3)P], phosphatidyl inositol 3,4-bisphosphate [PI(3,4)P2] and phosphatidyl inositol 3,4, 5-trisphosphate [PI(3,4,5)P3]. PI 3-kinase activation is regarded critical for the mitogenic potential of many growth factor receptors. In addition, the serine/threonine kinase AKT, key regulator of signalling and induction of metastasis which possesses a PI(3,4)P2/PI(3,4,5)P3-binding PH domain, requires PI 3-kinase activity for its activation by growth factor receptors [7]. Few studies showed that the PI3K/AKT pathway mediates cell proliferation and could regulate migration and adherents junctions [8]. PI-3K inhibition also reduced the expression of α5 integrin indicating a direct link between PI3K activity and α5 integrin expression [9].

Protein-protein interactions (PPIs) are a promising but challenging drug intervention target. The rational design of small-molecule inhibitors that mimic the chemical and physical properties of small clusters of key residues at the protein-protein interface is of significance. Identifying suitable interface residue clusters provides starting points for inhibitor design and supports an overall assessment of the susceptibility of PPIs to inhibition of small molecules. One approach to targeting PPIs is the rational design of small molecules that mimic the interaction at the protein-protein interface of a few key residues. Data helps us to understand potential biomarkers and therapeutic therapies in the fight against defined subpopulations of aggressive tumors and specific α5β1 integrin antagonists that may represent new potential therapeutic agents.

## Methodology

### Protein preparation and grid generation:

The 3D crystallized PI3-Kinase protein structure (PDB ID: 3HHM) from the protein databank (PDB) was used [10].The protein structure was refined using the protein prep [11] wizard to help transform the raw structure into a refined structure. Major preparation steps include hydrogen addition, removal of unwanted water molecules beyond 5Å optimization and minimization of the structure. Using the receptor grid generation, the active pocket in the prepared protein was freezed and it helps in converting the raw structure to a refined structure.

### Database preparation:

Zinc natural molecules database [12] has been retrieved, 2D to 3D molecular structure conversion and refining steps have been carried out using the Schrodinger software CANVAS module [13,14]. Conformations of molecules have also been generated through the Conf-Gen application [15].

### Pharmacophore hypothesis generation and database screening:

Two methodologies, structure-based drug design and ligand-based drug design, are well known for in silico screening approaches in the drug discovery pipeline. E-pharmacophore-based methodology combines both structural-based and ligand-based methods to screen the database of molecules [16,17].Using the crystal protein-ligand complex, a hypothesis was proposed, and the database was further checked using default values for the application.

### ADME and PAINS filters:

The screened molecules were further subjected to QikProp for ADME analysis [18,19]. Considering the main descriptors CNS as-2 (no activity against central nervous system), Human oral absorption to 100, Lipinski rule of five as zero parameters. The obtained ADME hits were followed with PAINS screening to remove the false positives.

### Binding studies:

Screened molecules have been docked at the active site of the PI3 kinase protein using the XP docking protocol [20-22] of the glide application. Selecting the protein grid file, scanning the molecules and setting up the docking protocol to XP with default values were completed using the application.Complexes were evaluated on the basis of binding modes between protein and ligand along with G-scores.

The G-scores were calculated based on the following formula

Glide Score = 0.065 * vdW + 0.130 * Coul + Lipo + Hbond + Metal + BuryP + RotB + Site

vdW-van der Waals energy, Coul-Coulomb energy, Lipo represents lipophilic term derived from hydrophobic grid potential, H bond-hydrogen-bond, Metal-metal-binding term, BuryP-buried polar groups, RotB-penalty for freezing rotatable bonds, and Site-polar interactions in the active site.

### Energy calculations:

The binding free energy of the final complexes was calculated using Prime/MM-GBSA [23] in the presence of the OPLS force field [24,25] in the VSGB solvent model.

dG Bind = Complex-Receptor-Ligand 

## Results and Discussion:

Molecular docking has become a standard tool in computational biology to predict the binding orientation of small molecule drug candidates to their protein targets in order to predict the affinity and activity of small molecules. Therefore, molecular docking plays an important role in the rational design of drugs. Compounds targeting PI3Kα have been studied. Non-selective first-generation PI3 K inhibitors such as wortmannin, LY 294002, quercetin, myricetin and stauroporin block both PI3Kα and PI3Kγ[26-27]. Therefore, it is of interest to define novel PI3-K-targeted inhibitors, a well-known kinase engaged in cell function such as growth, proliferation, differentiation, motility, survival and intracellular trafficking, all of which are linked to cancer development. Copanlisib, a broad PI3-K inhibitor, was used as a query structure for conformational similarity screening against database to find potential new PI3-K inhibitors.

**Figure 1:**
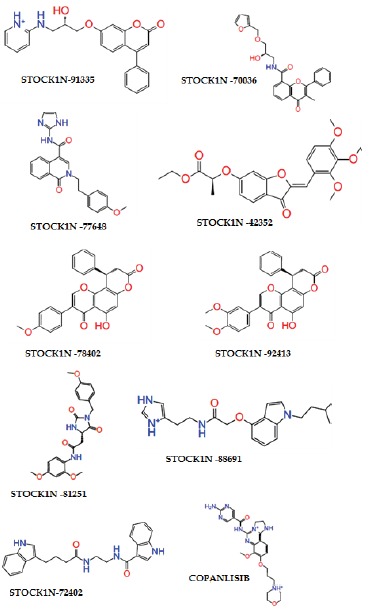
Molecular structures of selected nine molecules after screening

**Figure 2:**
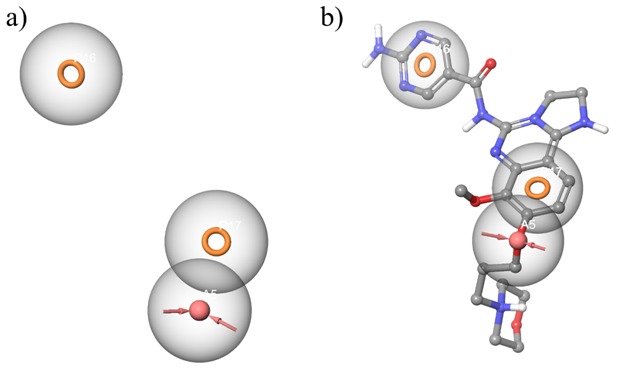
Illustration of hypothesis (a) RRA hypothesis; (b) Hypothesis alignment with the known inhibitor Copanlisib

PI3 kinase protein structural coordinates with a resolution of 2.8 Å from the protein database (PDB ID: 3HHM) were used. The protein was prepared to change the raw structure into the refined structure using the Protein Preparation Wizard. The receptor's active site was blocked by the position of the crystal ligand through the receptor grid generation. A functional kinase combination was needed to produce a pharmacophore theory. The famous copanlisib inhibitor was chosen as a template ligand in the LigPrep module. The prepared copanlisib and protein were paired with the Glide XP module of the Schrodinger software. The resulting pose viewer file was used for creating the hypothesis. The pharmacophore hypothesis calculates the pharmacophore properties of both the receptor and the ligand, generating a three-site RRA (R-Ring, A-Acceptor) hypothesis (Figure 2 A and B). The hypothesis has been imported into the Phase Database Screening Module and screened against the Natural Molecular Database. The total output of the drug screening was 1190 molecules. The molecules were screened with CNS as -2, rule 5 with 0-violations, 100% human oral absorption and, finally, rule 3 as nil, using the QiKProp module. Further testing of the 1190 molecules using the QikProp module took CNS (-2), Rule 5 (0), and human body absorption to 100 and ultimately Rule three to null. Based on these parameters, 9 molecules (Figure 1) out of 1190 molecules were found to satisfy the testing criteria (Table 1).

**Figure 3:**
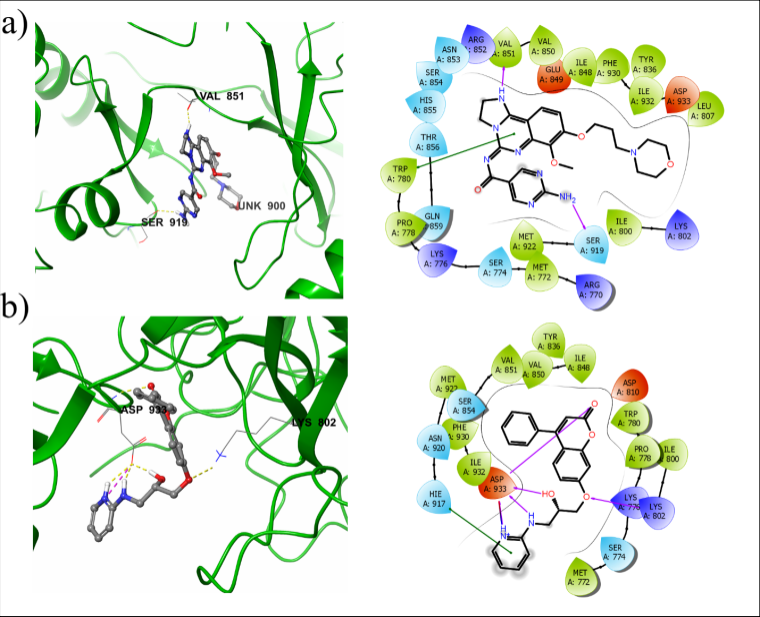
Binding interactions (a) Copanlisib; (b) STOCK1N-91335 with the residues present in the active site of PI3 Kinase

These molecules have been further screened using PAIN filters to remove the false positives from the 9 hits identified. All nine molecules were subjected to docking analysis using the Glide docking protocol XP. The results, the binding interactions between the molecules and the receptor, were compared with the known inhibitor. A G-score of-5.92 was noted for the known inhibitor by docking studies. Nine scores for molecular docking above the known inhibitor was selected for further studies. The interactions of molecules with the active protein site were further evaluated. Copanlisib, a well-known drug molecule, interacted with protein by forming hydrogen bonds with Val 851 and Ser 919. Hydrogen bonds were shared between the =O of Val 851 and the NH group present in the 5-member inhibitor ring (Figure 3A). A pi-pi stacking of the Trp 780 protein residue was observed along with the hydrogen bond. The known protein complex inhibitor maintained a G-score of-5.92. The top-ranked molecule STOCK1N-91355 was maintained with a complex binding affinity of five H-bonds. The hydrogen bonds shared between the residues and STOCK1N-91355 were as follows; -O of Asp 933 maintained four hydrogen bonds with STOCK1N-91355 at different positions (=O,-OH, NH and NH+) of the molecule and the last hydrogen bond was shared between the NH2 group Lys 802 and -O of the inhibitor (Figure 3B). The complex had a G-score of-10.21. The Protein and STOCK 1N-70036 complex had a G-score-9.38 with two hydrogen bonds. Residue Lys776 (NH+) made a hydrogen bond with -O in the five rings of STOCK1N-70036 and Lys802 (NH+) with -O connected to the five rings of the inhibitor. The protein–STOCK1N-77648 (G-score-8096) made hydrogen bonds with Lys 802, Val 851 and Ser 919 residues and one pi-pi interaction with Trp 780. The residual interaction profile with the inhibitor is as follows: Lys 802(NH3) with the inhibitor (-O) present in the six-member tail end ring; Val 851(NH) with the inhibitor (=O in the six-member ring) and Ser 919(=O) with NH present in the five-member inhibitor ring. The Trp 780 had a pi-pi interaction with the inhibitor in addition to H bonds. The binding interactions of STOCK1N-91335, STOCK1N-77648 and STOCK1N-42352 with PI3 kinase active site residues were shown in Figure 3. Hydrogen bonds between ligand and target have been reported in Table 2 Prime energy calculations show STOCK1N-91335 with an energy score of-62.10 kcal/mol having best binding features. Hits with-49.42 and-48.89 kcal/mol for STOKK1N-77648 and STOCK1N-42352, respectively is shown in Table 3.

**Figure 4:**
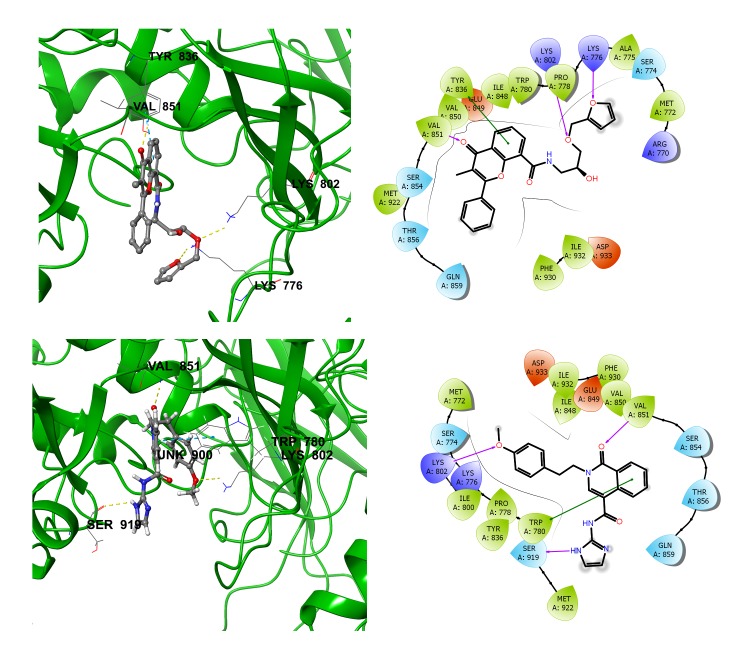
Binding interactions (a) STOCK1N-70036 (b) STOCK1N-77468 with the residues present in the active site of PI3 Kinase

Molecular docking and screening analysis data shows that STOCK1N-91335 molecule has the best binding features with the target protein. The molecule STOCK1N-70036 (Figure 4A) was second in the list. The third hit in the list is STOCK1N-77648 (Figure 4B). STOCK1N-77648 molecule made interactions to the protein residues, which are similar to the interactions made by the known inhibitor with best H-bonds pattern and G scores. The molecule STOCK1N-78402 also showed similar kind of interactions like that of the inhibitor but the complex G-score and the bind freenergy scores was low. The molecule STOCK1N-77648 was selected as the best inhibitor among the screened molecules for further consideration.

## Conclusion

PI3-K is a known target for anti-cancer drug development. Therefore, screening of small natural molecules database against the target for further consideration is of interest. We report STOCK1N-77648 with optimal binding atomic features with the target for further consideration through in vitro and in vivo validations.

## Figures and Tables

**Table 1:** ADME parameters of the selected 9 molecules

Molecule	CNS	Mol_mw	Percent Human oral absorption	Rule of five	Rule of three
STOCK1N-70036	-2	433.46	100	0	0
STOCK1N-81251	-2	413.42	100	0	0
STOCK1N-77648	-2	388.42	100	0	0
STOCK1N-91335	-2	388.42	100	0	0
STOCK1N-72042	-2	388.46	100	0	0
STOCK1N-78402	-2	414.41	100	0	0
STOCK1N-92413	-2	444.44	100	0	0
STOCK1N-42352	-2	428.43	100	0	0
STOCK1N-88691	-2	354.45	100	0	0

**Table 2:** Interaction profiles of the screened hits with the PI3 Kinase after docking studies

Complex	G-scores	Binding Interactions	
		Protein	Ligand
3HHM-78402	-8.3	Glu 849(OH)	=O
		Val 851(NH)	=O
		Lys 802(NH)	=O
		Trp 780(∏-∏)	
3HHM-42352	-8.21	Ser 774(OH)	=O
		Val 851(NH)	=O
		Arg 770(∏-cation)	
		Trp 780(∏-∏)	
3HHM-92413	-6.7	Lys 802(NH)	=O
		Glu 849(=O)	-OH
		Val 851(NH)	-OH
		Trp 780(∏-∏)	
3HHM-81251	-6.67	Lys 802(NH)	=O
		Tyr 836(OH)	=O
		Lys776(∏-cation)	
3HHM-88691	-6.34	Lys 802(NH)	-O
		Ser 919(=O)	NH
		Ser 774(OH)	=O
		Asp 933(-O)	NH
		Asp 933(SB)	

**Table 3:** Binding free energy calculations of the selected top three hits.

Molecule	Prime energy
	(Kcal/mol)
STOCK1N-91335	-62.1
STOCK1N-70036	-36.99
STOCK1N-77648	-49.42
STOCK1N-78402	-31.66
STOCK1N-42352	-48.89
STOCK1N-92413	-34.37
STOCK1N-81251	-35.07
STOCK1N-88691	-27.27
